# Optimizing Satellite Imagery Datasets for Enhanced Land/Water Segmentation

**DOI:** 10.3390/s25061793

**Published:** 2025-03-13

**Authors:** Marco Scarpetta, Luisa De Palma, Attilio Di Nisio, Maurizio Spadavecchia, Paolo Affuso, Nicola Giaquinto

**Affiliations:** Department of Electrical and Information Engineering, Polytechnic University of Bari, Via E. Orabona 4, 70125 Bari, Italy; marco.scarpetta@poliba.it (M.S.); attilio.dinisio@poliba.it (A.D.N.); maurizio.spadavecchia@poliba.it (M.S.); p.affuso@studenti.poliba.it (P.A.); nicola.giaquinto@poliba.it (N.G.)

**Keywords:** dataset quality evaluation, metrology for AI, NDWI, water detection, coastline monitoring, remote sensing, deep learning, satellite images

## Abstract

This paper presents an automated procedure for optimizing datasets used in land/water segmentation tasks with deep learning models. The proposed method employs the Normalized Difference Water Index (NDWI) with a variable threshold to automatically assess the quality of annotations associated with multispectral satellite images. By systematically identifying and excluding low-quality samples, the method enhances dataset quality and improves model performance. Experimental results on two different publicly available datasets—the SWED and SNOWED—demonstrate that deep learning models trained on optimized datasets outperform those trained on baseline datasets, achieving significant improvements in segmentation accuracy, with up to a 10% increase in mean intersection over union, despite a reduced dataset size. Therefore, the presented methodology is a promising scalable solution for improving the quality of datasets for environmental monitoring and other remote sensing applications.

## 1. Introduction

Over the last decades, there has been a remarkable growth of interest in environmental issues related to sustainability. Through advanced monitoring techniques, it is possible to obtain real-time data to mitigate risks, optimize performance, and ensure environmental compliance [1,2]. In this field, Earth observation is perceived as an important tool for environmental protection, sustainable development, and ecosystem conservation. In situ or aerial observations have traditionally provided high-resolution data over time, enabling effective and accurate environmental monitoring. However, these methods are often limited by high costs and significant time requirements. Over the past decade, the use of unmanned aerial vehicles (UAVs) and unmanned underwater vehicles (UUVs) has increased substantially, representing a major advancement over in situ analysis for remote sensing applications and other monitoring tasks, such as precision agriculture [3] or marine protection [4].

More recently, satellite imaging has been increasingly utilized across various research and technical domains, including environmental monitoring, urban planning, disaster management, and climate change assessment. One of its most critical applications is surface water monitoring, encompassing seas, lakes, and rivers, which are particularly vulnerable to the impacts of climate change [5]. In this context, satellite imagery plays a crucial role in tracking phenomena such as sea-level rise, coastal erosion, flooding, and droughts. By providing high-resolution, multi-temporal data, it enables researchers and policymakers to assess changes in water bodies, predict future trends, and implement mitigation strategies to address environmental challenges.

For the task of surface water monitoring, two main approaches can be employed: one based on pixel-level indexes and the other using deep learning models. Indexes leverage spectral bands from satellite imagery to enhance water-related signals while suppressing noise from vegetation or soil. Notable examples include the Normalized Difference Water Index (NDWI) [6], which employs the near-infrared (NIR) and green wavelengths to enhance water features, and the Modified Normalized Difference Water Index (MNDWI) [7], which replaces the NIR band with the shortwave-infrared band for improved performance in urban and turbid water areas. Other widely used indexes, such as the Automated Water Extraction Index (AWEI) [8] and the Water Ratio Index (WRI) [9], have been developed to improve accuracy under specific conditions, like shadowed or mixed land–water regions. These methods are widely used for surface water detection due to their simplicity, efficiency, and effectiveness in distinguishing water bodies from surrounding land features. Their advantages include low computational requirements, ease of implementation, and adaptability to various satellite platforms. However, they also have limitations, such as sensitivity to atmospheric effects, seasonal variations, and mixed pixels in areas with complex land–water boundaries. Additionally, since these methods operate on a pixel-by-pixel basis, they cannot leverage contextual information to improve classification in unclear or ambiguous regions. The main challenge with these approaches, however, is their reliance on threshold values to distinguish land from water, as no universally applicable threshold exists. This limitation complicates their use for large-scale or long-term spatial–temporal monitoring, especially in heterogeneous environments.

Data-driven monitoring techniques and deep learning models have increasingly replaced traditional index-based methods in remote sensing, effectively addressing many of their limitations [10,11,12]. In particular, deep convolutional neural networks (CNNs) have demonstrated strong performance across various remote sensing tasks, including image classification, object detection, change detection, and semantic segmentation. U-Net-based CNNs, for example, have achieved high accuracy in surface water segmentation, enabling detailed spatio-temporal monitoring of small water bodies such as rivers [13]. Additionally, vision transformer-based scene classification methods have been successfully applied to remote sensing tasks [14,15], further enhancing land cover classification accuracy on benchmark datasets [16,17].

A crucial aspect of AI systems is, however, their dependence on datasets, making the quality of these techniques heavily reliant on the quality of data used for training [18,19]. Even if all the engineering processes of ingesting, processing, and modeling work impeccably, data quality testing is essential at any stage of the data pipeline. This is particularly true for supervised learning-based AI, which utilizes datasets with labeled samples. Although datasets are critically important, their quality is rarely thoroughly evaluated in the field of environmental monitoring, and they are often used under the assumption that they have been accurately labeled [20].

For land/water segmentation, multiple datasets of annotated satellite images are available, with annotations generated through manual or semi-automatic procedures, resulting in varying levels of segmentation accuracy. The SWED [21] and SNOWED [22] provide Sentinel-2 Level 2A images containing water bodies with land/water segmentation annotations. In the SWED, labels are derived through a semi-automatic process in which multi-spectral index-based segmentation maps are manually refined. In contrast, SNOWED annotations are generated automatically using coastline measurements provided by NOAA. The S1S2-Water dataset [23] also contains Sentinel-2 land/water segmentation images, paired with Sentinel-1 images captured on the nearest available date. In this case, annotation follows a meticulous yet predominantly manual procedure: an initial segmentation map, obtained by applying a threshold to the NDWI, is manually refined in three subsequent steps by independent image interpretation experts.

Datasets based on very high-resolution (VHR) satellite imagery have also been developed. The dataset presented by Wieland et al. [24], derived from IKONOS imagery, follows a labeling approach similar to that of S1S2-Water. The GID dataset [25], which uses Gaofen-2 imagery, provides land cover classifications, including a water class, but does not specify the annotation methodology or any quality control measures. Other valuable datasets are also available, which focus specifically on flood monitoring, such as WorldFloods [26] and OMBRIA [27]. These datasets contain Sentinel-2 images capturing flooding events, with annotations derived from rapid mapping products created for disaster response. While these datasets are highly useful for emergency applications, their annotations are generated using semi-automatic methods and are not highly accurate, nor has their accuracy been quantified.

Collectively, the datasets described above reflect the effort invested in creating high-quality training data for deep learning models for land/water segmentation from satellite imagery. However, there remains a general shortage of annotated reference data for water segmentation, particularly of rigorously checked and validated samples [23]. The meticulous manual curation of some datasets, including validation by multiple independent experts [23,24], provides a strong indication of their quality; however, no objective, quantitative assessment of their accuracy is available. This highlights the need for an automated metrological approach to evaluate annotation quality, identify high-quality samples, and support the refinement of low-quality annotations. Such a method would enable the full utilization of valuable datasets for land/water segmentation. Since many existing datasets are affected by label errors and inconsistencies, improving their annotations could enhance their reliability and support the development of more accurate monitoring methods.

This research attempts to bridge this gap developed from our previous study [28], where we introduced the concept of automated dataset validation and presented the fundamentals of the algorithm and preliminary results. The proposed method makes use of the NDWI index with variable thresholds to evaluate annotation accuracy, providing a systematic framework for the quality analysis of these kinds of datasets.

To the best of our knowledge, no existing method in the literature systematically addresses this challenge. The only established quality assurance approaches rely on rigorous manual cross-checks conducted by multiple experts, which are time-consuming and labor-intensive. Our automated procedure, in contrast, leverages the NDWI to assess the consistency between the labels and their corresponding satellite images. Despite its limitations, the NDWI is strongly correlated with water presence and, when applied with an appropriate threshold, can generate labels that closely resemble the correct ones in the dataset, as evidenced by its use as the basis for annotation in several datasets [23,24]. The proposed approach enables a systematic and fully automated evaluation of each sample’s quality, leading to the creation of optimized dataset versions.

The paper is organized as follows. Section 2 describes the materials and methods, detailing the proposed dataset verification and optimization method, the evaluation procedure, and the characteristics of the datasets used to assess its effectiveness. Section 3 presents the results, including an analysis of dataset characteristics, the impact of the optimization process, and a performance comparison between deep learning models trained on baseline and optimized datasets. Finally, Section 4 provides the conclusions.

## 2. Materials and Methods

This section describes the proposed dataset optimization procedure and the methodology used to validate its effectiveness. Additionally, the two datasets on which the technique is applied are introduced.

### 2.1. Dataset Check and Optimization Method

The method proposed in this paper is designed to analyze large datasets of satellite images for land/water segmentation, aiming to assess the quality of each sample’s annotation and discard samples with low-quality or incorrect annotations. The core idea behind the quality assessment is to use index-based land/water segmentation to validate annotations: if a sample’s label is correct, there should exist a threshold value for the index map that produces a similar label. Conversely, if no NDWI threshold can generate a label with some similarity to the one in the dataset, the latter is likely of poor quality or entirely erroneous (e.g., with inverted classes).

In particular, the index employed is the NDWI, defined as follows:

(1) $$NDWI=\frac{\left(X_{G}-X_{NIR}\right)}{\left(X_{G}+X_{NIR}\right)}$$

where $X_{G}$ and $X_{NIR}$ are the reflectances at the green and NIR wavelengths, respectively. Other indexes mentioned in the Introduction can be used in place of the NDWI within the proposed procedure without altering its general framework. For instance, MNDWI or AWEI data could be employed, as they offer better performance in turbid water and shadowed scenes. However, these indexes require the short-wave infrared (SWIR) band for computation, which is not available in certain datasets, e.g., [24,25], making them unusable in those cases. Therefore, the NDWI was selected for this procedure, as it is the most established, widely used, and is more broadly computable due to the wider availability of the NIR band across multispectral satellite sensors. Nonetheless, the proposed approach remains flexible, and future developments could explore the integration of alternative indexes where suitable.

The similarity between the label and the segmentation map, obtained by applying a threshold to NDWI data, is quantified using the mean intersection over union (mIoU). The intersection over union (IoU) for a single class is defined as the ratio between the overlapping pixels of that class in the two maps being compared and the total pixels belonging to that class. The mIoU is then calculated as the average IoU across all classes (in this case, land and water), providing a comprehensive metric to evaluate the agreement between the maps being compared. Values of mIoU close to one indicate a high level of similarity between the two maps. Conversely, values around 0.5 likely reflect pixel misclassification for images presenting a significant class imbalance, where the dominant class achieves an IoU close to one, while the less prevalent class has an IoU near zero. Finally, values of mIoU close to zero represent the unusual scenario where one map is nearly the inverse of the other.

**Figure 1:**
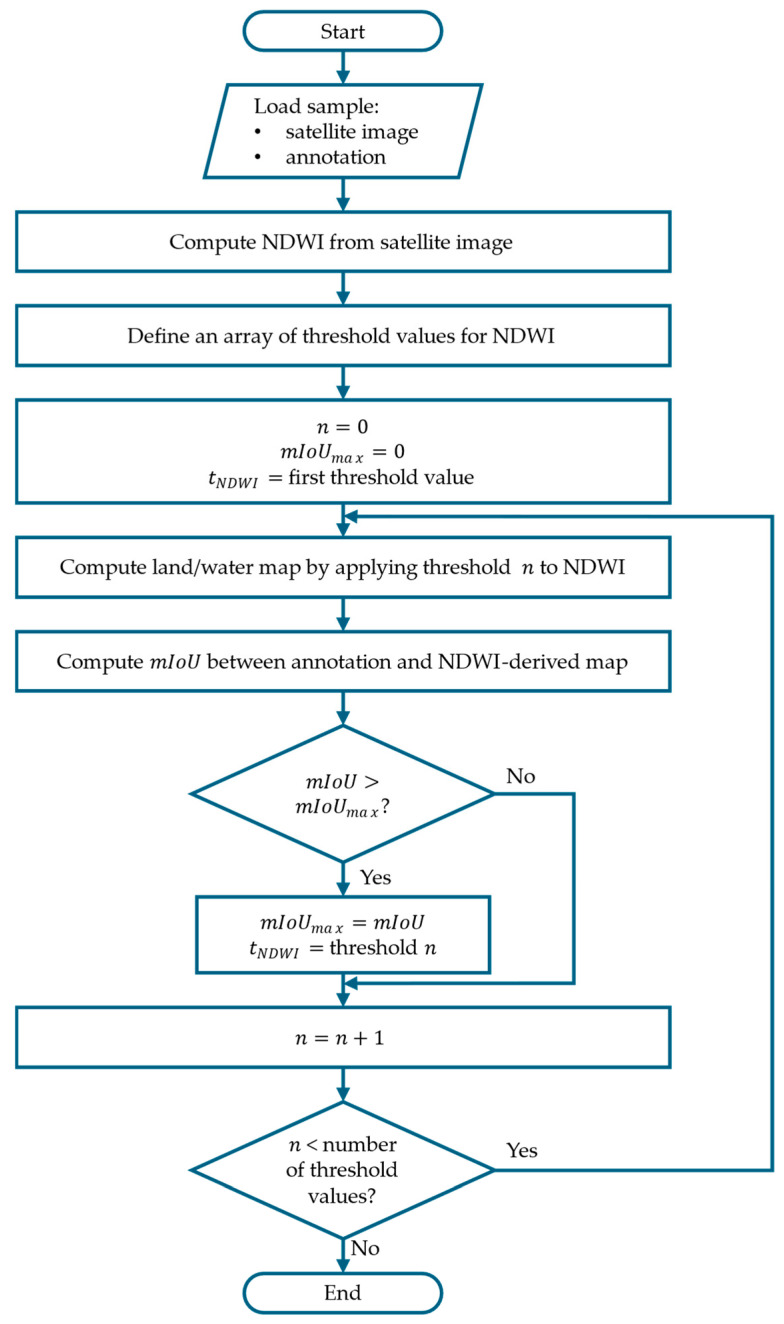
Flowchart of the algorithm employed to compute the optimal threshold for the NDWI map based on the annotation of the sample.

The optimal threshold value $t_{NDWI}$ for each sample, i.e., the threshold value that results in the maximum mIoU ($mIoU_{max}$) between the annotation and the NDWI-derived land/water segmentation, is determined using the iterative algorithm illustrated in Figure 1. To reduce the computational effort, the procedure is applied in three iterations, dynamically refining the search range of threshold values.

- First iteration (coarse search): 100 linearly spaced thresholds are calculated, ranging from the minimum to the maximum NDWI value for the sample. The coarse threshold estimation, $t_{NDWI}$, is then obtained by choosing the threshold that maximizes the mIoU.
- Second iteration (first refinement): a refined search is performed by computing an additional set of 100 linearly spaced thresholds within a narrower range. Specifically, this range extends from five values before to five values after $t_{NDWI}$ determined in the first iteration.
- Third iteration (second refinement): the same refinement process is applied again, but now within an even smaller range around the updated $t_{NDWI}$, obtained from the second iteration, further improving the threshold resolution.

After applying the procedure to the dataset under analysis, its samples can be ranked by $mIoU_{max}$ values. Those with $mIoU_{max}$ values that are too low can then be discarded or corrected, resulting in an optimized version of the dataset. More details about this operation are provided in the examples illustrated in Section 3.

### 2.2. Assessment of the Performance of the Datasets

To quantitatively assess the effectiveness of the proposed procedure, the optimized dataset was used to train a U-Net model for land/water segmentation. The performance of this model was then compared to that of a model trained on the baseline dataset, using various benchmark sets. One of these sets consists of 20% of the samples from the optimized dataset, which were consequently excluded from the training process of both models. The other test sets contain samples entirely independent of the dataset under analysis, allowing for an evaluation of the models’ generalization capabilities.

**Figure 2:**
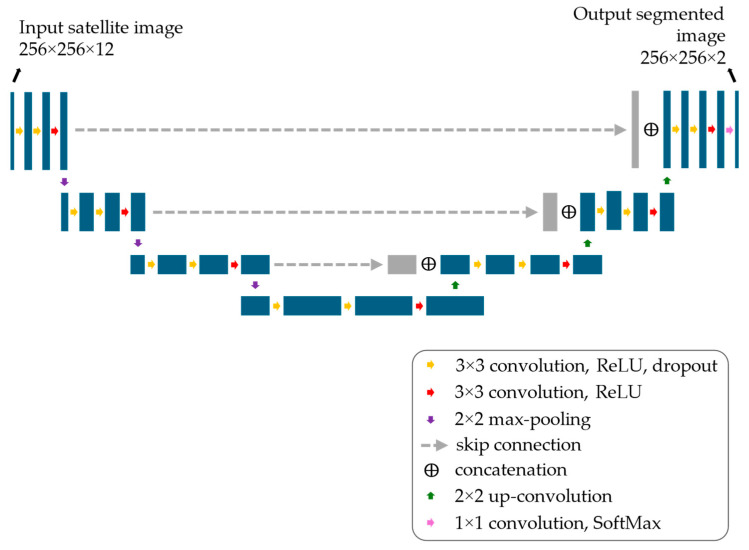
Architecture of the U-Net neural network model used to validate the optimized datasets.

A U-Net neural network [29] was chosen as the benchmark model, as this architecture has achieved remarkable success since its introduction and now serves as the foundation for many state-of-the-art image segmentation models [30,31,32]. The adopted U-Net model, previously employed in studies on satellite image segmentation [28,33], is illustrated in Figure 2. The contracting path consists of three consecutive convolutional blocks, each with 32 filters of 3 × 3 kernel size, ReLU activation, and dropout layers with a 20% rate for regularization. Finally, a max-pooling layer reduces spatial dimensions. This structure is repeated three times, doubling the number of filters at each step (from 32 to 128). These blocks lead to a bottleneck block composed of three convolutional layers with 256 filters, a 3 × 3 kernel size, and dropout layers with a 20% rate. The expanding path mirrors the contracting path but replaces standard convolutional layers with transposed convolutional layers. Skip connections link the contracting and expanding paths to preserve spatial information. Finally, the output convolutional layer, consisting of two 1 × 1 filters followed by the SoftMax activation function, produces the binary segmentation of the input image.

The U-Net neural network was trained separately on the optimized and baseline datasets, using the Adam optimizer [34] and binary cross-entropy loss function. After training, both models were evaluated on benchmark datasets based on mIoU and errors in estimating the water surface in each sample.

### 2.3. Datasets for Validation of the Optimization Procedure

The optimization procedure described in Section 2.1 can be applied to any dataset of multispectral satellite images for land/water segmentation, provided that the green and NIR bands are available for NDWI computation. This includes data from the two main public satellite missions, Landsat 8 and Sentinel-2. In this study, the procedure was tested using two datasets of Sentinel-2 images, which are described in the following sections.

#### 2.3.1. SWED

The Sentinel-2 Water Edges Dataset (SWED) [21] is a publicly available dataset of labeled Sentinel-2 satellite images, designed for remote sensing and deep learning applications. It was created by manually refining the approximate land/water segmentation labels generated through an automated process.

**Figure 3:**
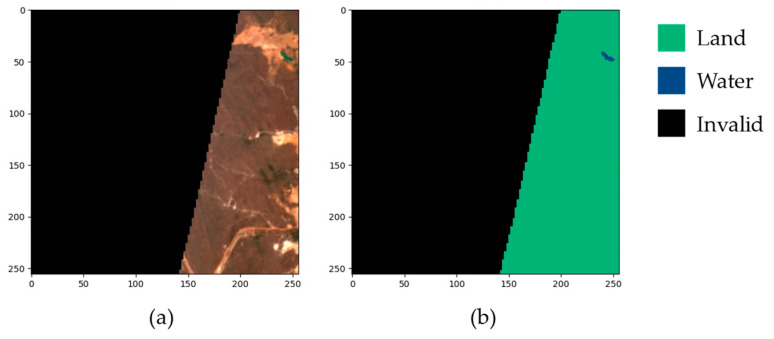
Example of a SWED sample with invalid label. (**a**) True color satellite image containing a region with missing data; (**b**) Corresponding label.

The SWED includes a primary dataset of 28,224 samples derived from 16 labeled Sentinel-2 tiles, along with a separate test set containing 98 samples. Each sample consists of a 256 × 256 pixel Sentinel-2 Level 2A image paired with its corresponding segmentation label. An automated analysis of the primary dataset identified 1703 samples with invalid labels, i.e., labels containing values other than 0 for land or 1 for water, such as the example shown in Figure 3.

The remaining 26,521 samples had valid labels, but 17,670 of these images contained only a single class (either land or water). While single-class samples can be useful for training a neural network to classify land and water images, they do not provide examples of coastlines, which are essential for teaching the network to distinguish between land and water pixels in transition regions. Moreover, these single-class samples can easily be obtained by selecting large regions of satellite images that contain only land or water. For these reasons, single-class samples were excluded from the subsequent analyses.

The SWED test set consists of 98 images, all with labels containing both land and water classes. As noted by the authors of [21], the labeling process for the test set was more rigorous than that of the primary dataset, ensuring that it provides a reliable benchmark for evaluating land/water segmentation models. Consequently, the SWED test set was used in this research as a reference for assessing the performance of models trained on the optimized dataset versus the baseline dataset.

#### 2.3.2. SNOWED

Similarly to the SWED, the “Sentinel2-NOAA Water Edges Dataset” (SNOWED) [22,35] consists of 4334 labeled images (each 256 × 256 pixels) derived from Sentinel-2 Level 2A data. Unlike the SWED, the SNOWED was created through an automated process that utilized certified, publicly available shoreline measurements from NOAA [36]. All samples encompass both classes (land and water) and were generated automatically, utilizing coastline measurements applied to satellite images captured on dates close to the corresponding NOAA data collection. This dataset can serve as either a standalone alternative to the SWED or in conjunction with it, allowing for the training of neural models using samples from both datasets.

From a conformity perspective, the SNOWED is free from significant human errors due to manual annotation. As a matter of fact, out of 4334 samples, none of them contain invalid or single-class labels. However, the dataset may still contain inconsistencies due to the inclusion, in the satellite images, of unregistered water bodies alongside those documented by NOAA. In addition, since it is not possible to obtain Sentinel-2 images captured precisely on the same date and time as NOAA measurements, variability and discrepancies between the labels and the actual water bodies depicted in the images are introduced.

## 3. Results and Discussion

### 3.1. Dataset Optimization Procedure

This section presents for the two considered datasets: (i) the results of a preliminary analysis of the samples in terms of water pixel percentage $w_{p}$, and (ii) the outcomes of the dataset optimization procedure presented in Section 2.1.

#### 3.1.1. SWED Optimization

**Figure 4:**
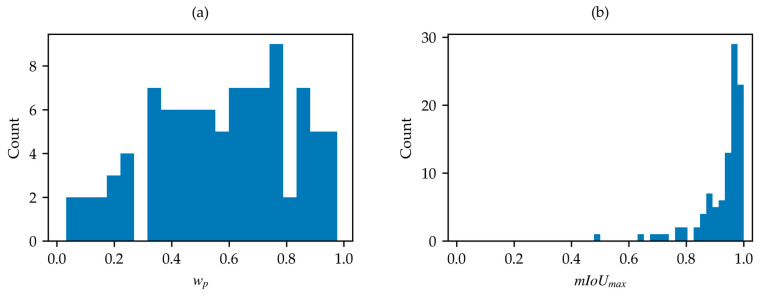
(**a**) Distribution of $w_{p}$ in SWED test set; (**b**) distribution of $mIoU_{max}$ in SWED test set.

Firstly, the SWED test set was analyzed due to its importance as a reference dataset. The water content percentage across the 98 samples reveals a minimum water percentage of 2.5% and a maximum of 97.7%, with the distribution shown in Figure 4a.

**Figure 5:**
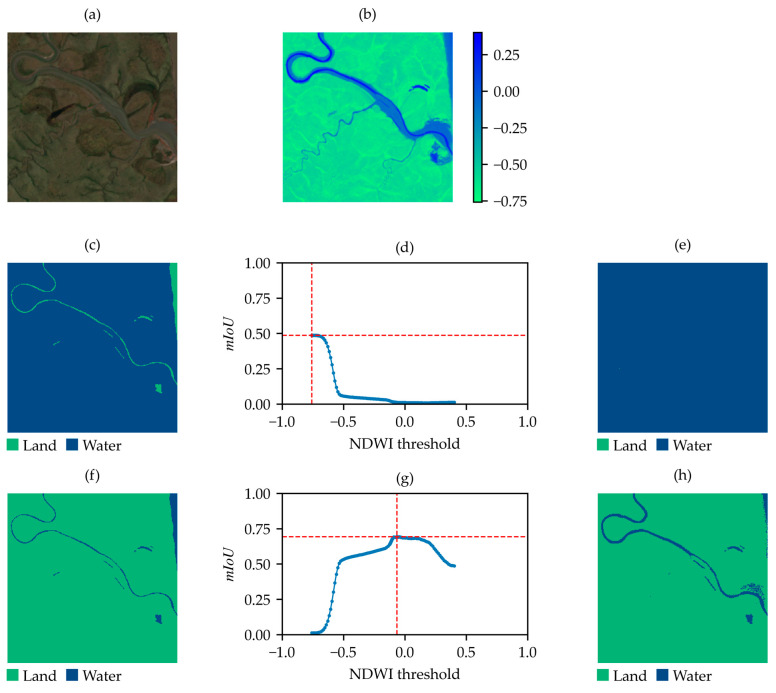
Outlier from the SWED test set with an inverted label. (**a**) True-color satellite image; (**b**) NDWI; (**c**) original label of the sample in the SWED test set; (**d**) mIoU values between the label in (**c**) and the NDWI-based binary map at varying thresholds; (**e**) binary map obtained by applying the threshold corresponding to the maximum mIoU in (**d**); (**f**) label with inverted classes; (**g**) mIoU values between the label in (**f**) and the NDWI-based binary map at varying thresholds; (**h**) binary map obtained by applying the threshold corresponding to the maximum mIoU in (**g**).

After the application of the optimization algorithm, the distribution of the $mIoU_{max}$ values for the 98 samples was also derived and shown in (Figure 4b). This distribution reveals a minimum $mIoU_{max}$ value of approximately 0.5 for a single sample, which is clearly anomalous compared to the remaining 97 samples, all of which have $mIoU_{max}$ values greater than 0.64. Inspection of the identified outlier, as shown in Figure 5, reveals that the label of this sample is inverted (water is marked as land, and vice versa) and the achievable $mIoU_{max}$ is about 0.49 (Figure 5c). After label correction, the computed $mIoU_{max}$ is 0.69 (Figure 5f).

**Figure 6:**
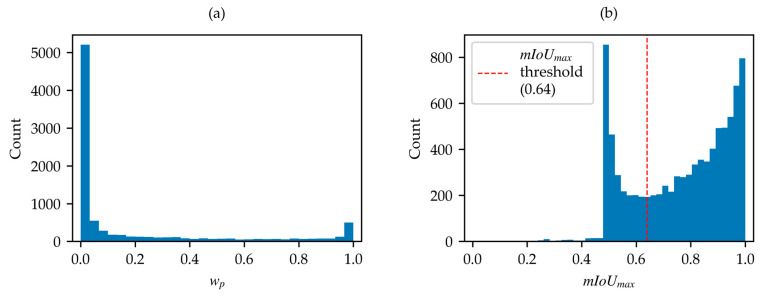
(**a**) Distribution of $w_{p}$ in SWED primary set; (**b**) distribution of $mIoU_{max}$ in SWED primary set.

Focusing the analysis on the SWED primary set, the distribution of $w_{p}$ for the 8851 samples with a valid label and containing both classes reveals a minimum close to 0% and a maximum of about 100%, with the distribution being skewed towards the extremes (Figure 6a). The distribution of the $mIoU_{max}$, obtained by applying the optimization algorithm on the samples of the SWED primary set, is also shown in Figure 6b.

The analysis of the distribution of $w_{p}$ and $mIoU_{max}$, together with the findings from the analysis of the SWED test set, leads to the definition of two criteria that must be met for sample acceptance:

(2) $$0.5\%<w_{p}<99.5\%$$

(3) $$mIoU_{max}>0.64$$

Criterion (2) was defined to further exclude not only samples with a single class but also those where one class is overwhelmingly dominant, with only a few pixels of the other class present. The water percentage limits were set using the findings from the SWED test set. The range of admissible values was widened (0.5–99.5% instead of 2.5–97.5%), to minimize the number of discarded samples.

Criterion (3) was established starting from the observation that samples with $mIoU_{max}\leq 0.5$ are certainly poor, while those with $mIoU_{max}$ close to 1 are certainly good. Therefore, the threshold must be set within this range. Analyzing the distribution of $mIoU_{max}$ values shown in Figure 6b, a minimum is observed at $mIoU_{max}\approx 0.64$, suggesting that this value could serve as a threshold to discriminate “bad samples” from “good samples”. This choice is further confirmed by the fact that, during the initial analysis of the SWED test set, no samples with $mIoU_{max}<0.64$ are found. Although this threshold may appear low, it is important to consider that experts can distinguish land and water in situations where the NDWI index may fail, such as in areas with ambiguous boundaries. As a result, $mIoU_{max}$ values close to 1 are not expected for all correctly labeled samples, as expert knowledge can identify features that the NDWI alone may not resolve.

By applying the proposed criteria to the 8851 samples of the SWED primary dataset containing both classes, we obtain the following results.

- In the whole dataset, 3688 samples do not meet criterion (2) (water percentage).
- In the remaining samples, 533 do not meet criterion (3) $\left(mIoU_{max}<0.64\right)$; however, swapping land and water classes, 124 of them achieve $mIoU_{max}>0.64$ and are therefore acceptable after this correction. Hence, a total of 533 − 124 = 409 samples must be discarded on the basis of criterion (3).
- After correcting the inverted labels, the total number of acceptable samples is 8851 − 3688 − 409 = 4754.

**Figure 7:**
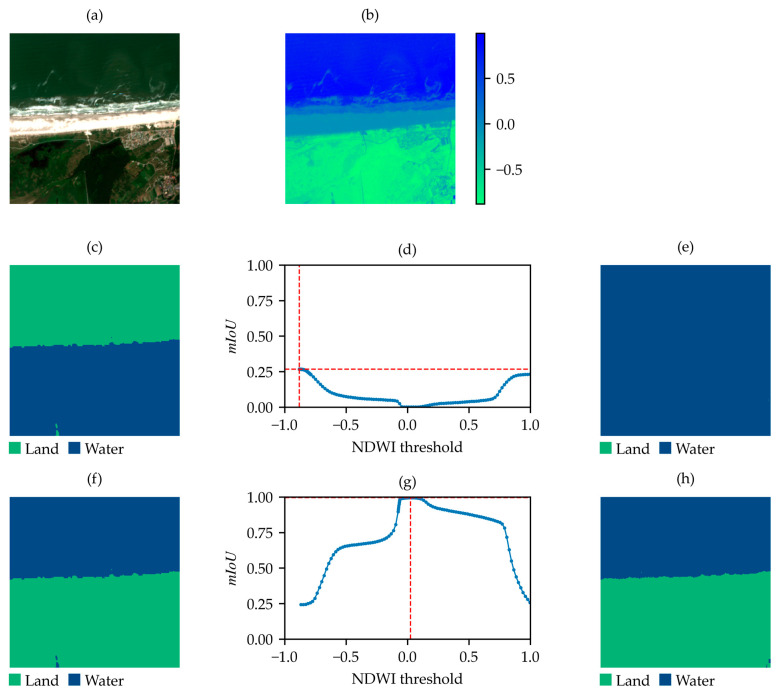
Sample from the SWED primary set with an inverted label. (**a**) True-color satellite image; (**b**) NDWI; (**c**) original label of the sample in SWED; (**d**) mIoU values between the label in (**c**) and the NDWI-based binary map at varying thresholds; (**e**) binary map obtained by applying the threshold corresponding to the maximum mIoU in (**d**); (**f**) label with inverted classes; (**g**) mIoU values between the label in (**f**) and the NDWI-based binary map at varying thresholds; (**h**) binary map obtained by applying the threshold corresponding to the maximum mIoU in (**g**).

The 124 samples with inverted labels are clearly the result of human errors during the labeling process. In Figure 7, an example is shown of a sample that requires inversion. As observed in Figure 7f, after the inversion of the classes, the sample can be considered acceptable.

#### 3.1.2. SNOWED Optimization

**Figure 8:**
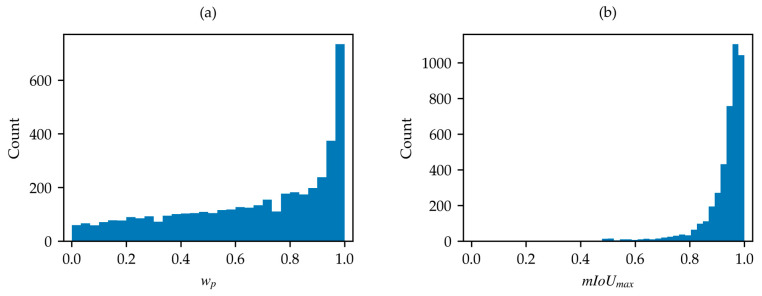
(**a**) Distribution of $w_{p}$ in SNOWED; (**b**) distribution of $mIoU_{max}$ in SNOWED.

The distributions of $w_{p}$ and $mIoU_{max}$ for the SNOWED are shown in Figure 8a and in Figure 8b, respectively. In this case, water percentage $w_{p}$ is more evenly distributed, though a shift toward higher values is noticeable. Conversely, the $mIoU_{max}$ distribution is strongly skewed toward higher values. These findings are consistent with the fact that the dataset is generated through an automated process based on high-accuracy coastline measurements. However, lower-quality samples are still present, making further optimization possible with the proposed algorithm. Its application results in the removal of 255 samples based on criterion (2), and 34 samples based on criterion (3), leaving 4045 acceptable samples. Notably, no samples requiring label inversion are found in this case.

**Figure 9:**
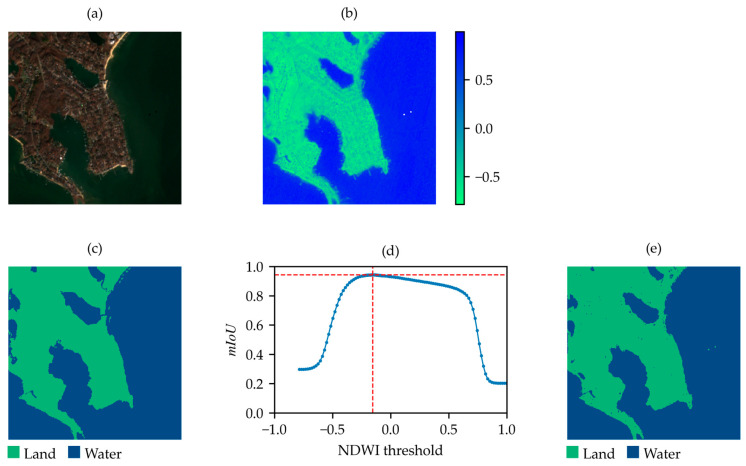
Example of acceptable SNOWED sample. (**a**) True-color satellite image; (**b**) NDWI; (**c**) label of the sample in SNOWED; (**d**) mIoU values between the label in (**c**) and the NDWI-based binary map at varying thresholds; (**e**) binary map obtained by applying the threshold corresponding to the maximum mIoU in (**d**).

**Figure 10:**
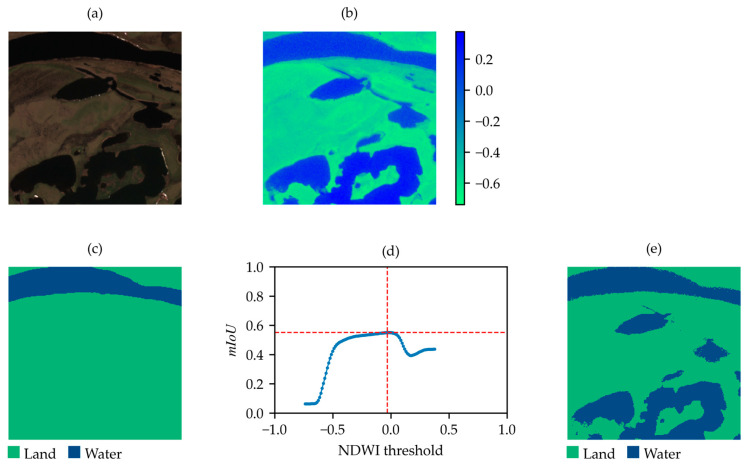
Example of non-acceptable SNOWED sample. (**a**) True-color satellite image; (**b**) NDWI; (**c**) label of the sample in SNOWED; (**d**) mIoU values between the label in (**c**) and the NDWI-based binary map at varying thresholds; (**e**) binary map obtained by applying the threshold corresponding to the maximum mIoU in (**d**).

Figure 9 and Figure 10 compare examples of acceptable (Figure 9) and non-acceptable (Figure 10) SNOWED images based on criterion (3). As shown, the non-acceptable sample includes water bodies that are not present in the label but are visible in the NDWI-based binary map corresponding to the $mIoU_{max}$. Therefore, the error in labeling is detected.

#### 3.1.3. Summary of the Dataset Optimization Procedure

The results of the dataset optimization procedure are summarized in Table 1, which reports the initial number of samples in each dataset, along with the number of non-acceptable and acceptable samples. Non-acceptable samples are categorized based on the cause of non-acceptance, with the numbers in each column indicating the samples remaining after applying the criteria in the preceding columns. Conversely, acceptable samples are divided into those used as-is and those accepted after inversion.

Analyzing the table, the SWED test set does not contain non-acceptable samples. This is expected, as it is a particularly curated dataset, and the selection criteria are specifically tailored to its characteristics. However, as previously noted, the dataset does include one sample that requires inversion. This underscores the fact that human errors in labeling are always possible, even when the utmost care is taken. The SWED, which was created through a semi-automatic procedure involving human intervention, contains non-acceptable samples in all categories as well as samples requiring inversion. Conversely, the SNOWED, which is based on an automated procedure, does not include samples with invalid classes, samples with only one class, or samples requiring inversion.

Detailed results from the application of the proposed algorithm to each sample in the SWED, SWED test set, and SNOWED are reported in Table S1.

### 3.2. Performance of the Optimized Datasets

The main characteristics of the datasets used for training and testing are summarized in Table 2. All models were tested on the SWED test set (*SWED_test*) and on two test sets whose samples are randomly extracted from the optimized versions of the SWED and SNOWED (*SWED_opt_test*, *SNOWED_opt_test*). The training datasets consist of the following: (i) the baseline SWED and SNOWED, excluding the samples used for the test sets *SWED_opt_test* and *SNOWED_opt_test*, and those with invalid labels that cannot be used for training; (ii) the optimized SWED and SNOWED, excluding the samples used for *SWED_opt_test* and *SNOWED_opt_test*. With this splitting of the data, each model was evaluated on three datasets, two of which are entirely independent on the one used for its training. Models trained on the baseline SWED and SNOWED were compared to those trained on optimized versions of the datasets.

The results of the performance evaluation of the trained models are reported in Table 3 for the SWED and in Table 4 for the SNOWED. All the models were tested using the three test datasets described in Table 2, with mIoU and the root mean squared error of water surface estimation ($RMSE_{w}$) serving as performance metrics.

For the SWED training datasets, both metrics (mIoU and $RMSE_{w}$) show significant improvement when using the optimized dataset, compared to the baseline, across all three test sets. Notably, an increase of over 10% is observed in the mIoU for the SWED test set and the SNOWED validation set, which are the two external (independent) test datasets in this case. This improvement is particularly noteworthy, considering that the optimized dataset *SWED_opt_train* is only about 15% the size of the baseline dataset *SWED_train*.

For the SNOWED training dataset, most metrics improve, with the exception of the mIoU for the SNOWED validation set, which exhibits a slight decrease of 0.1%, but with an improvement of 8% in $RMSE_{w}$ (from 1560 to 1432 pixels). While performance improvements for the SNOWED are observable, they are less pronounced than those obtained for the SWED. This difference can be attributed to the extent of changes between the baseline and optimized datasets. Specifically, the optimized SNOWED differs from the baseline for only 289 out of 3525 samples, resulting in smaller performance differences. In contrast, the SWED sees substantial modifications, with 21,767 out of 25,570 samples removed, leading to more significant improvements.

These values confirm the superiority of the models trained on the optimized datasets, compared to the models trained on baseline datasets, not only on the test sets extracted from the optimized datasets (*SWED_opt_test*, *SNOWED_opt_test*) but also on the *SWED_test* set, which is particularly challenging to predict with high accuracy.

**Figure 11:**
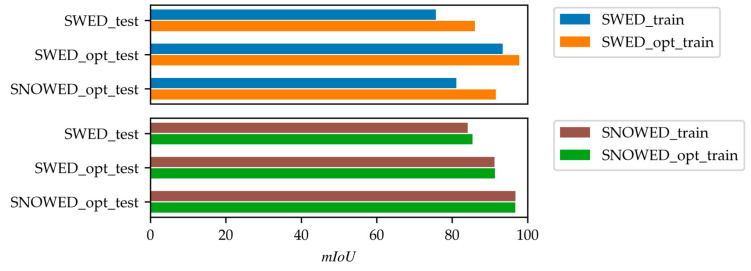
Performance comparison of models trained on the optimized SWED and SNOWED versus their respective original datasets, measured in terms of mIoU.

**Figure 12:**
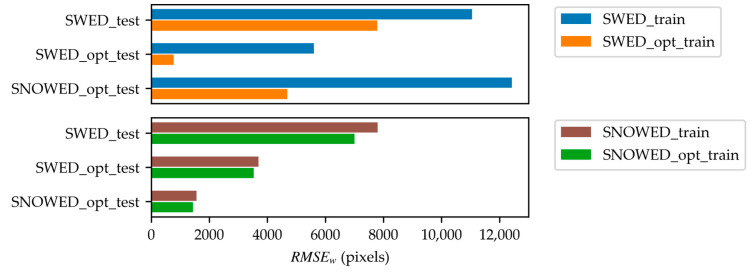
Performance comparison of models trained on the optimized SWED and SNOWED versus their respective original datasets, measured in terms of $RMSE_{w}$.

The results presented in Table 3 and Table 4 are also illustrated in Figure 11 and Figure 12 for clarity. Specifically, Figure 11 compares the performance of the original and optimized versions of the SWED and SNOWED across the three test datasets in terms of mIoU, while Figure 12 presents their performance in terms of $RMSE_{w}$.

## 4. Conclusions

This study proposes an optimization procedure to enhance the quality of datasets used in land/water segmentation tasks with deep learning models. The method was applied to two publicly available datasets, the SWED and SNOWED, which contain multispectral Sentinel-2 satellite images and their corresponding segmentation labels. By analyzing key dataset characteristics, criteria have been established to filter out low-quality samples, refine training data, and improve model performance.

Experimental results demonstrate that models trained on the optimized datasets achieve significant improvements over those trained on the baseline datasets. Notably, for the SWED, an increase of more than 10% in mean intersection over union was observed on independent test sets, despite the optimized dataset being only a fraction of the size of its original counterpart. These findings highlight the effectiveness of dataset curation in improving segmentation accuracy, while also reducing computational requirements.

The proposed optimization procedure is generalizable and can be applied to other multispectral datasets where land/water segmentation is required. Beyond the selection process performed in this study, samples not meeting the defined quality criteria after simple corrections (like label inversion) could be manually corrected using NDWI-based binary maps, obtained by applying the optimal threshold value. This approach would facilitate the expansion of high-quality samples within the dataset while maintaining label reliability.

The presented methodology highlights the importance of developing automated methods to evaluate and improve dataset quality, rather than solely focusing on constructing larger datasets, as a key factor for advancing research in environmental monitoring and remote sensing. More broadly, this research underscores the importance of metrologically rigorous review processes for datasets employed for training deep learning models and emphasizes the need for more robust approaches to ensure label accuracy in automatic labeling procedures.

## Figures and Tables

**Table 1:** Summary of the dataset optimization procedure.

Dataset Name	Total Number of Samples	Non-Acceptable Samples					Acceptable Samples		
		Contain Invalid Classes	Contain Only One Class	Criterion (2) Not Met	Criterion (3) Not Met	Total	As Is	After Inversion	Total
SWED Test Set	98	0	0	0	0	0	97	1	98
SWED	28,224	1703	17,670	3688	409	23,470	4630	124	4754
SNOWED	4334	0	0	255	34	289	4045	0	4045

**Table 2:** Datasets used for training and testing the U-Net models.

Usage	Name	Composition	Number of Samples
Testing	SWED_test	SWED test set	98
	SWED_opt_test	20% of optimized SWED	951
	SNOWED_opt_test	20% of optimized SNOWED	809
Training	SWED_train	All SWED samples (except for those with invalid labels and those included in the test set)	25,570
	SWED_opt_train	80% of optimized SWED	3803
	SNOWED_train	All SNOWED samples (except for those included in the test set)	3525
	SNOWED_opt_train	80% of optimized SNOWED	3236

**Table 3:** Performance results on the optimized SWED vs. the baseline SWED.

Training Dataset	Test Dataset					
	SWED_Test		SWED_Opt_Test		SNOWED_Opt_Test	
	mIoU	$RMSE_{w}$ (Pixels)	mIoU	$RMSE_{w}$ (Pixels)	mIoU	$RMSE_{w}$ (Pixels)
SWED_train	75.7%	11,051	93.4%	5603	81.1%	12,425
SWED_opt_train	**86.0%**	**7788**	**97.8%**	**775**	**91.6%**	**4683**

**Table 4:** Performance results on the optimized SNOWED vs. the baseline SNOWED.

Training Dataset	Test Dataset					
	SWED_Test		SWED_Opt_Test		SNOWED_Opt_Test	
	mIoU	$RMSE_{w}$ (Pixels)	mIoU	$RMSE_{w}$ (Pixels)	mIoU	$RMSE_{w}$ (Pixels)
SNOWED_train	84.1%	7801	91.2%	3693	**96.8%**	1560
SNOWED_opt_train	**85.4%**	**7003**	**91.3%**	**3528**	96.7%	**1432**

## Data Availability

The publicly available SWED used in this study can be found at: https://openmldata.ukho.gov.uk/ (accessed on 6 February 2025). The publicly available SNOWED used in this study can be found at: https://zenodo.org/records/8112715 (accessed on 6 February 2025).

## Supplementary Materials

The following supporting information can be downloaded at: https://www.mdpi.com/article/10.3390/s25061793/s1, Table S1: Results from the application of the proposed algorithm to each sample in the SWED, SWED test set, and SNOWED.
